# Supplementary material for: The interactome of intact mitochondria by cross-linking mass spectrometry provides evidence for coexisting respiratory supercomplexes
Source: Mol Cell Proteomics. 2017 Dec 8;17(2):216–32. doi: 10.1074/mcp.RA117.000470 (PMC5795388; doi:10.1074/mcp.RA117.000470)
Supplement: Supplemental Data [file supp_RA117.000470_133922_0_supp_23978_fzffwf.zip › spectra_annotation/mito_DR_spectra_annotation/442-1-29-1-7-1.pdf]

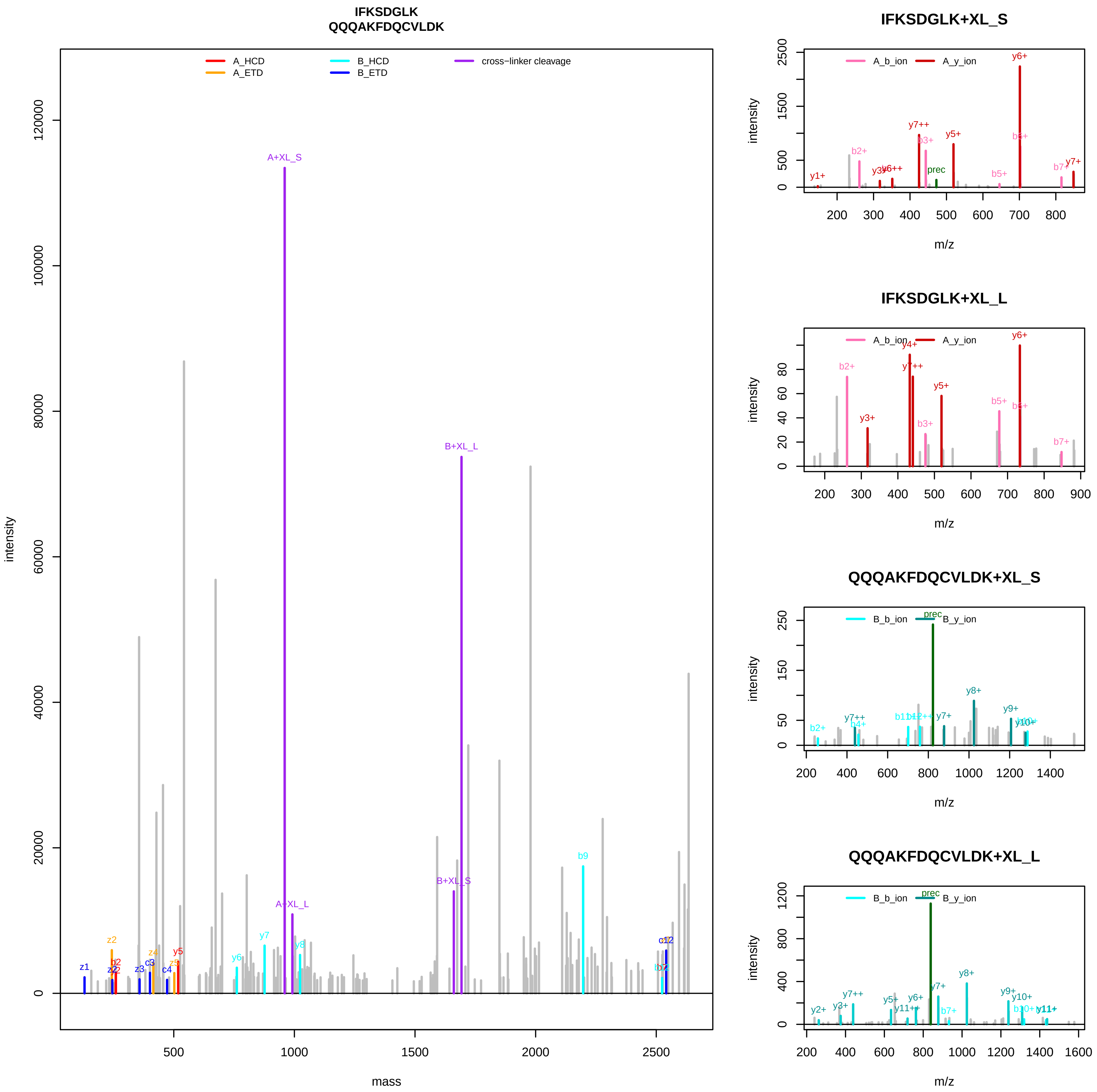
IFKSDGLK
QQQAKFDQCVLDK
A_HCD
A_ETD
B_HCD
B_ETD
cross−linker cleavage
A+XL_S
B+XL_L
B+XL_S
A+XL_L
z1
z2
z2
b2
z3
c3
z4
c4
z5
y5
y6
y7
y8
b9
c12
b12
intensity
mass
0
20000
40000
60000
80000
100000
120000
500
1000
1500
2000
2500
IFKSDGLK+XL_S
A_b_ion
A_y_ion
y1+
b2+
y3+
y6++
y7++
b3+
prec
y5+
b5+
b6+
y6+
b7+
y7+
intensity
m/z
IFKSDGLK+XL_L
y4+
y7++
y5+
y3+
b3+
b5+
b6+
b7+
QQQAKFDQCVLDK+XL_S
B_b_ion
B_y_ion
prec
b2+
y7++
b4+
b11++
b12++
y7+
y8+
y9+
y10+
QQQAKFDQCVLDK+XL_L
y2+
y3+
y7++
y5+
y11++
y6+
b7+
y7+
y8+
y9+
y10+
b10+
y11+
